# Obesity Correlates With Pronounced Aberrant Innate Immune Responses in Hospitalized Aged COVID-19 Patients

**DOI:** 10.3389/fimmu.2021.760288

**Published:** 2021-10-11

**Authors:** Michael Z. Zulu, Suhas Sureshchandra, Amanda N. Pinski, Brianna Doratt, Weining Shen, Ilhem Messaoudi

**Affiliations:** ^1^ Department of Molecular Biology and Biochemistry, University of California, Irvine, Irvine, CA, United States; ^2^ Institute for Immunology, University of California, Irvine, Irvine, CA, United States; ^3^ Department of Statistics, University of California, Irvine, Irvine, CA, United States; ^4^ Center for Virus Research, University of California, Irvine, Irvine, CA, United States

**Keywords:** obesity, aging, SARS-CoV-2, COVID-19, innate immunity, monocytes, cytokines

## Abstract

Both age and obesity are leading risk factors for severe coronavirus disease 2019 (COVID-19), which is caused by severe acute respiratory syndrome coronavirus 2 (SARS-CoV-2). Specifically, although most infections occur in individuals under the age of 55 years, 95% of hospitalizations, admissions to the intensive care unit, and deaths occur in those over the age of 55 years. Moreover, hospitalized COVID-19 patients have a higher prevalence of obesity. It is generally believed that chronic low-grade inflammation and dysregulated innate and adaptive immune responses that are associated with aging and obesity are responsible for this elevated risk of severe disease. However, the impact of advanced age and obesity on the host response to SARS-CoV-2 infection remains poorly defined. In this study, we assessed changes in the concentration of soluble immune mediators, IgG antibody titers, frequency of circulating immune cells, and cytokine responses to mitogen stimulation as a function of BMI and age. We detected significant negative correlations between BMI and myeloid immune cell subsets that were more pronounced in aged patients. Similarly, inflammatory cytokine production by monocytes was also negatively correlated with BMI in aged patients. These data suggest that the BMI-dependent impact on host response to SARS-CoV-2 is more pronounced on innate responses of aged patients.

## Introduction

The global pandemic of coronavirus diseases (COVID-19) caused by the severe acute respiratory syndrome coronavirus 2 (SARS-CoV-2) continues to have a significant impact on human health and daily life activities (1). As of August 2021, there were almost 37 million infections and over 600,000 deaths due to SARS-COV-2 in the United States alone (2). Most infected individuals (40-80%) experience an asymptomatic or mild disease (3, 4) with the remaining 20% of cases requiring medical attention or hospitalization are over-represented by elderly patients (>60 years) and those with pre-existing comorbidities, notably obesity, diabetes, and cardiovascular diseases (5–19). Indeed, obese individuals are reported to be 46% more likely to contract SARS-CoV-2 infection, 100% more likely to be hospitalized upon infection, and 48% more likely to succumb to infection due to severe COVID-19 compared to non-obese individuals (20). On the other hand, case fatality rate increases progressively with age with 15.4% of the deaths occurring in those 50-64 years of age, 21.4% in those 65-75 years of age and 30.7% in those over 85 years of age (21). Studies have also reported that individuals over the age of 65 years are reported to be 60% more likely to be hospitalized upon infection and 95% more likely to succumb to infection than patients younger than 40 years of age (22). Patients with severe COVID-19 present with respiratory failure, dyspnea, pneumonia, acute respiratory distress syndrome (ARDS), and long-term complications that can culminate in death (20, 23–26).

Obesity is associated with chronic low-grade inflammation (27), high leptin and C-reactive protein (CRP) (28), and altered immune responses (29). Indeed, dysregulated immune responses to various pathogens is well-documented in obese individuals, including an exacerbated TNF-α and IL-6 response that is accompanied by suppressed T-cell responses to bacterial and viral infections (30, 31). Obesity is associated with reduced immune response to influenza infection and vaccination (32) as well as disruptions of lymphoid tissue integrity in addition to alterations in leukocyte development, phenotypes and functions (33). However, the mechanisms that fully elucidate how obesity exacerbates COVID-19 remain elusive. Angiotensin-converting enzyme 2 (ACE2), a cellular receptor for SARS-CoV-2 is highly expressed in adipose tissue, which could potentially explain the increased susceptibility of obese individuals to SARS-CoV-2 infection (34–36). Obesity in the elderly population is extremely underestimated since body fat accumulates in parallel with the muscle mass decline and vertebral compression which results in the reduction of height, a phenomenon known as sarcopenic obesity (37). Age-related changes in soluble immune mediators, and both innate and adaptive immune responses are known to influence susceptibility to infections, disease progression and clinical outcomes as well as response to therapeutics and vaccines (38, 39). Nonetheless, age-related immune changes associated with greater severity, and adverse outcomes of COVID-19 in obese young and aged patients remain relatively unknown.

Numerous studies have reported complex immune dysregulation with severe COVID-19 in both young and aged patients. One of the hallmarks is a cytokine storm characterized by high blood concentration of pro-inflammatory cytokines such as IL-6, IL-1β and TNF-α, as well as chemokines such monocytes chemoattractant protein-1 (MCP-1) and interferon-inducible protein 1- (IP-10) (40–42). Moreover, several changes in antigen presenting cells (APCs) have been described including a pronounced decrease of HLA-DR expression on monocytes and dendritic cells (DC) (43, 44); a decrease in the frequency of total DC (45); and an increase in non-classical monocytes (45, 46) compared to patients with mild disease and healthy controls. Some studies (45) reported no changes in the number of B cells and NK cells while others described a drastic decrease in all major lymphocyte subsets (CD4+ T cells, CD8+ T cells, NK cells and B cells) in severe COVID-19 cases compared to mild cases (40). Other studies reported that patients with high BMI generally have a higher frequency of regulatory immune cells compared to pro-inflammatory cells in the blood (47). However, there is a paucity of studies examining the impact and interaction of obesity and age on innate and adaptive immune responses in severe COVID-19. Therefore, we investigated the impact of BMI on immune pathogenesis of COVID-19 in young and aged hospitalized patients with severe COVID-19.

## Results

### BMI Associated Aberrant Innate Immune Responses Are Marked on Aged COVID-19 Patients

**Figure 1:**
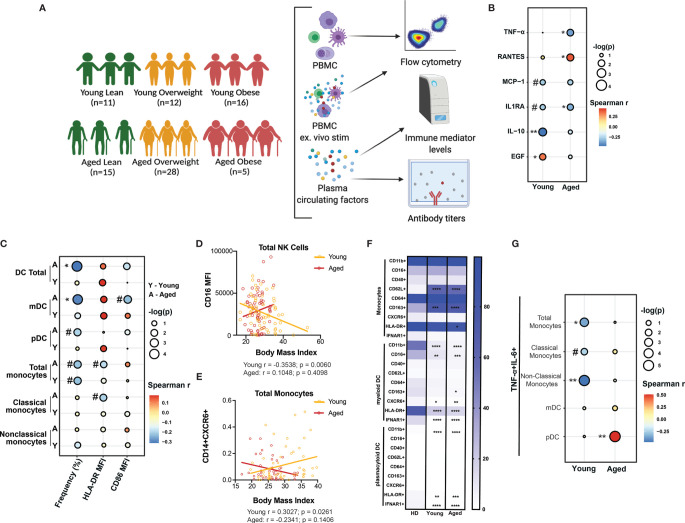
Body mass index (BMI)-dependent aberrations in soluble immune mediators and innate immunity in young and aged COVID-19 patients. **(A)** Experimental design for the study. Blood was collected from 39 young (Lean=11, Overweight=12 and Obese=16) (<60 years of age) and 48 aged (Lean=11, Overweight=28 and Obese=5) hospitalized COVID-19 patients (>60 years of age). Immune phenotypes of PBMC and concentration of soluble immune mediators in plasma were determined by flow cytometry and Luminex, respectively. Serological responses to SARS-CoV-2 were measured by ELISA. A subset of PBMC were *ex vivo* stimulated with anti-CD3/CD28 dynabeads or bacterial agonist cocktail (Pam3CSK4, LPS, and FSL-I) to measure T cells and monocytes cytokine secretion, respectively, by flow cytometry. **(B)** Linear regression analysis of the concentration (pg/mL) of soluble immune mediators (cytokines, chemokines, and growth factors) that showed a significant or a trend of positive or negative correlation with BMI in both young and aged patients. **(C)** Linear regression analysis of the frequency of dendritic cell (DC) and monocyte subsets, expression of MHC class II molecule HLA-DR, and co-stimulatory molecule, CD86 with patient BMI in young and aged hospitalized COVID-19 patients. **(D)** Linear regression analysis of the expression of CD16 (FcγRIII) on total CD56+ Natural killer (NK) cells with patient BMI in young and aged groups. **(E)** Linear regression analysis of the frequency of CD14+CXCR6+ monocytes with patient BMI in young and aged groups. **(F)** Differences in the phenotype of monocytes and DC subsets from young and aged COVID-19 patients compared to healthy donors. **(G)** Linear regression analysis of the dual expression of pro-inflammatory cytokines (IL-6 and TNF-α) by monocytes and DC subsets in response to 8-hr *ex vivo* stimulation with bacterial agonists (Pam3CSK4, LPS, and FSL-I) with patient BMI. #p < 0.12, *p < 0.05, **p < 0.01, ***p < 001 and ****p < 0001.

To investigate the impact of BMI on pathogenesis of COVID-19 in young (<60 years of age) and aged (>60 years of age) hospitalized patients, we performed phenotypic and functional assays using blood samples from 39 young and 48 aged patients who were classified based on body mass index (BMI) into lean (BMI ≤ 24.9 kg/m^2^), overweight (25-29.9 kg/m^2^) and obese (≥30 kg/m^2^) (**Figure 1A**). Additional patient characteristics are shown in **Supplementary Table 1**. First, we measured the levels of circulating soluble immune mediators using Luminex assay. There was a significant increase in the concentration of RANTES [CCL5, a chemoattractant for memory T cells and monocytes (48)] in COVID-19 patients compared to healthy donors. We also observed a significant increase in the concentration of the monocyte chemoattractant protein-1 (MCP-1) and epidermal growth factor [EGF, which plays a key role in host response to coronaviruses and is implicated in lung disease induced by highly pathogenic respiratory viruses (49)] in young COVID-19 patients only whereas a trend towards high concentration of EGF was observed in aged patients (p=0.0848) (**Supplementary Figure 1A**). Stratifying these analyses by BMI in each group showed a significantly high concentration of RANTES in overweight and obese aged patients while MCP-1 was significantly lower in overweight young and obese aged patients compared to their lean and overweight counterparts (**Supplementary Figure 1B**). To assess the effect of obesity on systemic inflammation, we performed a linear regression analysis of the concentration of circulating soluble immune mediators (cytokines, chemokines, and growth factors) with patient BMI in young and aged patients (**Supplementary Table 2**). BMI negatively and positively correlated with IL-10 (anti-inflammatory cytokine) and EGF levels, respectively, in young patients (**Figure 1B**). BMI negatively correlated with TNFα and IL-1RA (pro-inflammatory and anti-inflammatory mediators respectively) levels in aged patients (**Figure 1B**). In contrast, levels of RANTES (Spearman r = 0.3999; p = 0.0191) positively correlated with BMI in aged patients. Moreover, modest negative correlation between BMI and IL1RA (Spearman r = -0.2704; p = 0.1162) and MCP-1 (Spearman r = -0.2759; p = 0.1143) was observed in young patients (**Figure 1B**). These results suggest that obesity further exacerbates the balance between anti and pro-inflammatory cytokines especially in aged patients.

We then assessed the association between BMI and the frequency of circulating dendritic cells (DC) and monocyte subsets by flow cytometry (**Supplementary Figure 1C** and **Supplementary Table 3**). Previous studies have reported reduced frequencies of DC subsets but increased frequency of monocytes in patients with severe COVID-19 (43, 44). The frequency of total DC (Spearman r = -0.3122; p = 0.012) and myeloid (m)DCs (Spearman r = -0.2954; p = 0.0169) was significantly negatively correlated with BMI, while that of plasmacytoid (p)DCs showed modest negative correlation (Spearman r = -0.1995; p = 0.1112) in aged patients (**Figure 1C**). A modest negative correlation between the frequency of total monocytes and BMI was observed in young (Spearman r = -0.2248; p = 0.0959) and aged (Spearman r = -0.2195; p = 0.0978) patients. Additionally, the expression level of HLA-DR (defined by mean fluorescence intensity; MFI) on total (Spearman r = -0.1883; p = 0.1407) and classical monocytes (Spearman r = -0.2028; p = 0.1268) as well as the MFI of CD86 on mDC (Spearman r = -0.2303; p = 0.082) was also modestly negatively correlated with BMI in aged patients (**Figure 1C**). Although no significant correlation was observed between BMI and the frequency of natural killer (NK) cells or their subsets, (**Supplementary Figure 1D**), the expression level of CD16 (FcγRIII) on NK cells was negatively correlated with BMI in young patients only (Spearman r = -0.3538; p=0.0060) (**Figure 1D**). Collectively, these observations suggest that disruptions in myeloid cells frequencies and their effector functions (such as antigen presentation and antibody dependent cell cytotoxicity) caused by SARS-CoV-2 infection are further exacerbated by obesity.

Next, we compared expression of markers associated with cell activation (CD40), maturation (CD16), homing (CXCR6), tissue recruitment (CD62L), and polarization (CD163) on monocytes and DC subsets in young and aged COVID-19 patients relative to healthy donors. Levels of monocytes expressing CD62L and CD163 in both young and aged COVID-19 patients were significantly higher compared to healthy donors (**Figure 1F**). In line with what we (46) and others (44, 45, 50) had previously reported, MHC Class II molecule, HLA-DR was significantly downregulated on total monocytes, mDC, and pDC of both young and aged patients compared to healthy donors (**Figure 1F**). Additionally, CD11b and IFNAR1 expression was significantly downregulated on mDC and pDC, while CD16 expression was also reduced on mDC in both young and aged patients compared to healthy donors (**Figure 1F**). Next, we determined the correlation between patient BMI and the frequency of monocytes and DC subsets expressing these markers using a linear regression analysis (**Supplementary Figure 1E** and **Supplementary Table 4**). Only the frequency of CD14+CXCR6+ monocytes was positively correlated with BMI (Spearman r = 0.3027; p=0.0261) in young patients (**Figure 1E**). These data are consistent with recent reports of monocytes’ skewing towards regulatory phenotype with SARS-CoV-2 infection (46).

Next, we determined the correlation between BMI and the frequency of myeloid cells (monocytes and DC subsets) secreting pro-inflammatory cytokines (TNF-α and IL-6) in response to a bacterial agonists cocktail consisting of Pam3CSK4 (TLR1/2), FSL-1 (TLR2/6) and LPS (TLR4) (**Supplementary Figure 1F** and **Supplementary Table 5**). The frequency of responding (secreting both TNF-α and IL-6) total monocytes (Spearman r = -0.3573; p = 0.0351), and non-classical monocytes (Spearman r = -0.4692; p = 0.0059) negatively correlated with BMI in young patients only, whereas that of responding classical monocytes showed a modest negative correlation (Spearman r = -0.2463; p = 0.1538) (**Figure 1G**). No significant correlation was observed between the frequency of responding DC subsets (mDC and pDC) and BMI in young patients. In contrast, the frequency of pDC secreting both TNF-α and IL-6 positively correlated (Spearman r = 0.51; p = 0.0047) with BMI in aged patients only (**Figure 1G**). Taken together, these data suggest that the dysregulation of innate immune response to severe COVID-19 is more pronounced in obese aged patients.

### Differential Effects of BMI on Adaptive Immunity in Young and Aged Patients

**Figure 2:**
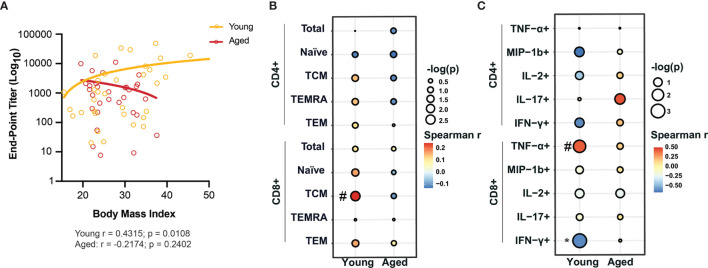
BMI-dependent alterations of adaptive immunity in young and aged COVID-19 patients. **(A)** Linear regression analysis of IgG end-point titers against nucleocapsid protein (NP) with BMI in young and aged hospitalized COVID-19 patients. **(B)** Linear regression analysis of the frequency of CD4+ and CD8+ T cell subsets with BMI in young and aged hospitalized COVID-19 patients. **(C)** Linear regression analysis of the frequency of cytokine producing CD4+ and CD8+ T cells in response to *ex vivo* anti-CD3/CD28 stimulation with BMI in young and aged hospitalized COVID-19 patients. #p < 0.12, *p < 0.05, **p < 0.01, ***p < 001 and ****p < 0001.

To assess how BMI modulates the adaptive arm of the immune system, we profiled frequency of lymphocyte subsets including proliferating lymphocytes, *ex vivo* T cell responses to anti-CD3/CD28 stimulation, and Immunoglobulin G (IgG) antibody titers against SARS-CoV-2. There were no significant correlations with BMI and frequency of any of the B cell subsets (total, naïve, marginal-zone-like (MZ-like) and other B cells) in both young and aged patients (**Supplementary Figure 2A** and **Supplementary Table 6**). However, we observed a modest positive correlation between the frequency of proliferating other B cells (Spearman r = 0.2118; p = 0.1014) and BMI in the aged group (**Supplementary Figure 2B** and **Supplementary Table 6**). Interestingly, obese young patients had significantly higher IgG titers against SARS-CoV-2 nucleoprotein (NP) compared to young lean patients; but no other differences in SARS-CoV-2-specific IgG endpoint titers between lean, overweight and obese patients in both young and aged patient groups were observed (**Supplementary Figure 2C**). In young patients, the end-point titers of IgG against SARS-CoV-2 nucleoprotein (NP) positively correlated with BMI (Spearman r = 0.4315; p = 0.0108) (**Figure 2A**), whereas no association was observed for RBD IgG titers (**Supplementary Figure 2D** and **Supplementary Table 7**) in both young and aged patients. These data suggest that antibody responses against NP may be an indicative of disease severity.

No significant correlation between BMI and frequency of CD4+ and CD8+ T cell subsets in either young or aged patients were observed except for a positive association between frequency of CD8+ central memory (CD8+ TCM) and BMI (Spearman r = 0.2445; p = 0.0776) in young patients (**Figure 2B**, **Supplementary Figure 2E** and **Supplementary Table 6**). Additionally, the frequency of proliferating CD4+ TCM positively correlated with BMI (Spearman r = 0.3158; p = 0.0132) in aged patients (**Supplementary Figure 2F**). Finally, we also measured the frequency of CD4+ and CD8+ T cells that secreted various cytokines in response to anti-CD3/CD28 stimulation (**Supplementary Figure 2G**, **Supplementary Table 8**). Linear regression analyses revealed a significant negative correlation of the frequency of CD8+ T cells secreting IFN-γ (Spearman r = -0.6182; p = 0.0478) while that of CD8+ T cells secreting TNF-α showed a modest negative correlation (Spearman r = 0.4229; p = 0.0917) with BMI in young patients only (**Figure 2C**). These observations suggest that obesity is associated with a dysregulated production of antiviral cytokines by T cells.

## Discussion

Age, obesity and associated co-morbidities are considered crucial predictors of adverse COVID-19 outcomes (51–53). Severe COVID-19 in the elderly population is associated with higher rates of acute respiratory distress syndrome (ARDS), lymphopenia, and cardiovascular complications (54–56). Recent studies reported higher odds ratio of severe pneumonia due to obesity in men, older adults, and those with diabetes (57); a high prevalence of obesity in severe COVID-19 cases that required invasive mechanical ventilation (IMV) (58) and that the need for IMV significantly correlated with male sex and BMI, independent of age, diabetes and hypertension (59). However, the immune mechanisms associated with increased susceptibility to severe COVID-19 in aged patients and those with obesity are yet fully elucidated.

Here, we investigated BMI-associated changes in various immune parameters in young (<60 years of age) and aged (>60 years of age) hospitalized patients. Our analysis of circulating immune mediators did not reveal the cytokine storm that has been widely reported as a hallmark of severe COVID-19 (42, 60–62). This difference could be due to the introduction of corticosteroids as standard of care for COVID-19 patients which has been shown to dampen the cytokine storm associated with severe disease (63–65). Despite the dampened systemic inflammatory response, and as reported previously (46, 66), we observed a high concentration of RANTES/CCL5, a chemokine that binds CC chemokine receptor 5 (CCR5) to regulate trafficking and effector functions of memory and effector T cells and macrophages (67) that was more pronounced in aged COVID-19 patients. This observation is in line with previous studies that reported a 3-5-fold increase in RANTES/in mild/moderate patients and >100-fold in critical ones (68). Disruption of the CCL5/RANTES-CCR5 pathways was recently shown to restore immune homeostasis and reduce plasma viral load in COVID-19 critical patients (68, 69). Our analysis further revealed a significant positive correlation between BMI and RANTES/CCL5 in aged patients only. Therefore, these data suggest that RANTES/CCL5 may be driven by BMI and that the use of corticosteroids which became standard of care before our study samples were collected may not fully disrupt the production of RANTES/CCL5-especially in aged patients. Our analyses also revealed a negative correlation of the concentration of the pro-inflammatory cytokines MCP-1/CCL2 concentration which has been associated with disease severity (66, 70, 71); was significantly higher only in aged patients. Additionally, levels of EGF were increased in COVID-19 patients, especially aged patients, compared to healthy donors and positively correlated with BMI in young COVID-19 patients. Growth factors are involved in the process of viral infection (72) and EGF was shown to increase the incidence of fibrosis following SARS-infection (49). Interestingly, levels of anti-inflammatory factors IL-10 and IL-1RA were modestly negatively correlated with BMI in young patients, which could lead to increased levels of systemic inflammation. Overall, our data suggest that immune modulators may be less effective in combating the inflammatory immune response associated with severe COVID-19 the elderly population with high BMI.

We also observed that frequency of DC and monocytes were negatively correlated with BMI in aged COVID-19 patients. Monocytes, mDC and pDC are critical for antiviral immune responses (73, 74). Defects in frequency, phenotype, and function of these cells have been previously reported in severe COVID-19 patients (44, 46, 50, 75, 76). Moreover, we and others have also reported a decrease in the expression of HLA-DR and CD86 on various subsets of monocytes and DC, which could further impact their ability to initiate T cell responses (43, 44, 46). Interestingly, the frequency of total and non-classical monocytes that expressed inflammatory cytokines, tumor necrosis factor-alpha (TNF-α) and interleukin-6 (IL-6) in response to bacterial agonists negatively correlated with BMI in young patients whereas in aged patients. We recently reported decreased responses by monocytes and DC from CVID-19 patients to bacterial ligands (46). A further reduction with BMI could signal increased susceptibility to bacterial secondary infections which have been reported as a stronger predictor for death than influenza amongst severe COVID-19 patients (77).

In contrast, the frequency of plasmacytoid dendritic cells (pDC) that expressed both TNF-α and IL-6 positively correlated with BMI in aged patients only. pDC are specialized cells of the innate immune system known for their natural ability to produce type I interferons (78) and sensing viral RNA and DNA *via* toll-like receptor-7 (TLR-7) and TLR-9 (79). Therefore, our data suggests that there is an increased activation of pDC due to SARS-CoV-2 infection leading to a robust type I IFN-dependent immunity against secondary infection (i.e., bacterial infection) that may further exacerbate the cytokine storm leading to adverse disease outcomes in aged patients (80). We also report that the expression level of CD16 (FcγRIII) on NK cells is negatively correlated with BMI in young patients only. The downregulation of FcγRIII on peripheral blood NK cells has been previously reported as a virus-induced NK cell-mediated immunity evasion strategy of HIV infection (81). This mechanism has also been implicated in SARS-CoV-2 infection (82). FcγRIII is crucial for antibody-dependent cellular cytotoxicity (83), therefore, our data suggest that functional NK cells decrease as a function of BMI in young COVID-19 patients.

Monocytes and dendritic cells are mononuclear phagocytes (MNPs) in peripheral blood that are critical mediators of innate and adaptive immune responses during viral infection (84). Dysregulation of these cells is associated with COVID-19 severity (85). We report an increased frequency of monocytes expressing CD62L and CD163 in the blood from both young and aged COVID-19 patients. CD62L expression is associated with the recruitment of monocytes to tissue from blood during inflammation (86) while CD163, is a monocyte/macrophage scavenger-receptor and a marker of monocyte/macrophage activation (87). Therefore, these data suggest that the recruitment and activation of monocytes from blood to the upper respiratory tract during SARS-CoV-2 infection may not be affected by age. However, we also noted that the frequency of monocytes expressing lung homing marker, CXCR6 (88, 89) positively correlated with BMI in young patients while a trend towards negative correlation was observed in aged patients. Collectively, these data suggest an age-dependent differential effect of BMI on immune cell activation and recruitment during SARS-CoV-2 infection.

SARS-CoV-2 infection elicits robust humoral immunity (90). However, the effect of obesity and age on its durability remains ill-defined. We report a significant positive correlation between end-point titers of IgG against SARS-CoV-2 nucleoprotein (NP) in young patients only, whereas no significant correlation was observed for RBD IgG in both young and aged patients. Indeed, SARS-CoV-2 antibody responses were recently reported to correlate with disease severity (91). Interestingly, obesity has been reported to impair humoral immunity through the suppression of B cell development and antibody production in vaccine studies (92). We also report a significant positive correlation between the frequency of central memory CD8+ T cells (CD8+ TCM) and BMI in young patients only; and increased proliferation of central memory CD4+ T cells (CD4+ TCM) in aged patients only, these differences may be independent of COVID-19 since obesity has been reported to greatly increase memory T cell frequencies (93). In response to CD3/CD28 stimulation, the secretion of TNF-α by CD8+ T cells trended towards positive correlation with BMI while IFN-γ secretion by CD8+ T cells significantly negatively correlated with BMI in young patients, suggesting an impairment of CD8+ T cells antiviral with obesity.

In summary, we assessed correlations between patient BMI and the concentration of immune mediators, humoral immunity and the phenotype and function of immune cells to determine the effect of obesity on the molecular pathogenesis of severe COVID-19 in the peripheral blood of young and aged hospitalized patients receiving corticosteroids treatment. We report that obesity correlates with aberrant innate immune responses that is more pronounced in aged patients. One of the major limitations of the study is the lack of careful measurements of adiposity in the elderly. Additionally, we did not have access to information pertaining to the treatments the patients received during their hospital stay. Future studies should address the impact of age and obesity on efficacy and durability of immune responses to COVID-19 vaccines.

## Materials and Methods

### Study Participants and Experimental Design

This study was approved by University of California Irvine Institutional Review Board (HS# 2012-8716). Remnant blood samples from a total of 39 young (11 lean, 12 overweight and 16 obese) and 48 aged (15 lean, 28 overweight and 5 obese) COVID19 patients admitted to the University of California Irvine Medical Center (UCIMC) were obtained through the COVID-19 biospecimen bank between January and March 2021. Samples were stratified by age (<60 categorized as young and ≥ 60 categorized as aged) and BMI (≤ 24.9 categorized as lean; 25-29.9 categorized as overweight, and > 29.9 categorized as obese).

### Plasma and Peripheral Blood Mononuclear (PBMC) Isolation

Peripheral blood mononuclear cells (PBMCs) and blood plasma samples were isolated after whole blood centrifugation 1200 g for 10 minutes at room temperature in SepMate tubes (STEMCELL Technologies). Blood plasma was stored at -80^0^C until analysis. PBMC were cryopreserved using 10% DMSO/FBS and Mr. Frosty Freezing containers (Thermo Fisher Scientific) at -80C then transferred to a cryogenic tube and stored in liquid nitrogen until analysis.

### Luminex

Immune mediators such as cytokines including, IFN-α2, IFN-γ, TNF-α, IL-1α, IL-1β, IL-1ra, IL-2, IL-4, IL-5, IL-6, IL-7, IL-8, IL-10, IL-12 (p40), IL-12(p70), IL-13, IL15, and IL-17A; chemokines such IP-10, MCP-1, Eotaxin/CCL11, MIP-1α, MIP-1β; and growth factors such G-CSF,EGF and VEGF were measured using the Human Cytokine/Chemokine Magnetic Bead Panel (HCYTMAG-60K-PX29) (MilliporeSigma, MA, USA). Plasma samples were thawed and diluted per manufacturer’s instructions and run-in duplicates on the Magpix instrument (Luminex, Austin, TX). Data were fit using a 5P-logistic regression on xPONENT software (version 7.0c).

### Antibody ELISA

Clear 96-well, high-binding polystyrene ELISA plates were coated with 100uL/well of 500 ng/mL SARS-CoV-2 Spike-protein (S) (GenScrip) or 1 μg/mL SARS-CoV-2 Nucleocapsid Protein (NP) (GenScrip) in PBMS overnight at 4C. Plates were brought to RT, unbound antigen removed by flicking and blocked by adding 200uL/well of blocking buffer for 1h at RT. Heat inactivated plasma (1:50 in blocking buffer) was added to the well and incubated for 90 minutes at RT. Responses were visualized by adding HRP-anti-human IgG (BD Pharmingen) to the wells. Reaction was visualized using o-Phenylenediamine dihydrochloride (ThermoFischer Scientific) diluted with hydrogen peroxide (H_2_O_2_). Reaction was stopped with 50uL/well of 1M HCL. ODs were read at 490nm on a Victor3™ Multilabel plate reader (Perkin Elmer).

### Peripheral Blood Immune Cell Phenotyping

Frozen PBMCs were thawed, washed, and counted before staining with Ghost Dye viability dye (TONBO biosciences) to delineate between live and dead cells. We then proceeded to staining for surface markers, permeabilized, fixed and then stained intracellularly using the Foxp3/Transcription Factor Staining Buffer Kit (TONBO biosciences) as per manufacturer’s instruction. We measured changes in the frequency and activation of circulating innate immune cells using the following set of antibodies: CD3 (SP34, BD Pharmingen) and CD20 (2H7, Biolegend) for the exclusion of T & B lymphocytes, respectively. We further stained for CD56 (BV711, Biolegend), CD57 (HNK-1, Biolegend), KLRG1 (SA231A2, Biolegend) CD16 (3G8, Biolegend), CD14 (M5E2, Biolegend), HLA-DR (L243, Biolegend), CD11c (3.9, ThermoFisher Scientific), CD123 (6H6, Biolegend) and CD86 (IT2.2, Biolegend) to detect changes in the peripheral blood frequency and activation of Natural Killer (NK) cells, Monocytes, myeloid (mDCs) and plasmacytoid dendritic cells (pDCs). To measure changes in the frequency and proliferation of adaptive immune cells, we used the following set of antibodies: CD4 (OKT4, Biolegend), CD8b (2ST8.5H7, Beckman Coulter), CD45RA (HI100, TONBO Biosciences), CCR7 (G043H7, BD Biosciences), CD19 (HIB19, Biolegend), IgD (IA6-2, Biolegend), CD27 (M-T271, Biolegend), KLRG1 (SA231A2, Biolegend) and PD-1 (Eh12.2h7, Biolegend). Following permeabilization, cells were fixed and then stained with proliferation marker Ki-67 (B56), and cytotoxic molecule, Granzyme B (QA16A02). All samples were acquired in batches on the Attune NxT software v2.7.0 on the Attune NxT acoustic focusing cytometer (Life Technologies) and FCS files were analyzed using FlowJo v10 (TreeStar, Ashland, OR USA).

### Myeloid Cell Phenotyping

1x10^6^ cryopreserved PBMC were thawed then washed twice in FACS buffer and surface stained using the following antibody cocktail: CD14 (M5E2, Biolegend), CD16 (3G8, Biolegend), CD11b (ICRF44, Biolegend), HLA-DR (L243, Biolegend), CD40 (5C3, BD Pharmingen), CD62L (DREG-56, BD Pharmingen), CD64 (10.1, Biolegend), CD163 (GHI/61, Biolegend), CXCR6 (K041E5, Biolegend), IFNAR (85228, R&D Systems), CD11c (3.9, ThermoFisher Scientific) and CD123 (6H6, Biolegend) for the detection of various monocytic and dendritic cell subsets. Samples were then acquired on the Attune NxT acoustic focusing cytometer (Life Technologies). Data were analyzed using FlowJo v10 (TreeStar, Ashland, OR USA).

### Myeloid Cell Response to Bacterial Ligands

1x10^6^ cryopreserved PBMC were thawed, washed, and then stimulated with a bacterial agonist cocktail or left unstimulated. The bacterial agonist cocktail consisted of a combination of 2ug/mL Pam3CSK4 (TLR1/2 agonist, InvivoGen), 1ug/mL FSL-1 (TLR2/6 agonist, Sigma Aldrich), and 1ug/mL LPS (TLR4 agonist from *E. coli* 0111: B4, InvivoGen). Samples were cultured for 1 hour before adding protein transport inhibitor (Brefeldin A) and incubated for an additional 7 hours at 37C. Cells were then washed twice in FACS buffer and surface stained using the following antibody cocktail – CD14 (M5E2, Biolegend) and HLA-DR (L243, Biolegend) for 30 minutes at 4C. Stained cells were then fixed and permeabilized using Fixation buffer (Biolegend) and incubated overnight with a cocktail of intracellular antibodies – IL-6 (MQ2-6A3, Biolegend), and TNFɑ (Mab11, eBioScience). Samples were then acquired on the Attune NxT acoustic focusing cytometer (Life Technologies). Data were analyzed using FlowJo v10 (TreeStar, Ashland, OR USA).

### T Cell Intracellular Cytokine Staining

1x10^6^ cryopreserved PBMC were thawed, washed, and then stimulated for 16 hours at 37C in the presence or absence of anti-CD3/CD28 dynabeads per manufacturer’s instructions (ThermoFisher Sceintific); Brefeldin A (Sigma Aldrich, St. Louis, MO) was added after 1 incubation. Cells were stained for surface markers CD4 (OKT4, Biolegend) and CD8b (2ST8.5H7, Beckman Coulter), fixed, permeabilized, and then stained intracellularly for IFN-γ (4S.B3, Biolegend), TNF-α (Mab11, ThermoFischer Scientific), MIP-β (D21-1351, BD Pharmingen), IL-2 (MQ1-17H12, Biolegend) and IL-17 (eBio64DEC17, ThermoFisher Scientific). Samples were then acquired on the Attune NxT acoustic focusing cytometer (Life Technologies). Data were analyzed using FlowJo v10 (TreeStar, Ashland, OR USA).

### Statistical Analysis

Data sets were first tested for normality. An ordinary one-way analysis of variance (ANOVA) test was used to compare readouts from young and aged study participants to the healthy donors (HD); and to compare readouts between lean, overweight and obese participants in both young and aged groups. Linear regression analysis was used to compare significant shifts in curve over horizontal line, with spearman correlation coefficient reported for each group. P-values less than or equal to 0.05 were considered statistically significant. While P-values between 0.05 and 0.12 are reported as trending patterns. Graphs were created using R (version 4.0.1) and GraphPad Prism version 8.4.3 (GraphPad Software, LLC).

## Data Availability Statement

The original contributions presented in the study are included in the article/**Supplementary Material**. Further inquiries can be directed to the corresponding author.

## Ethics Statement

The studies involving human participants were reviewed and approved by University of California Irvine Institutional Review Board. The patients/participants provided their written informed consent to participate in this study.

## Author Contributions

MZ, SS, and IM conceived and designed the experiments. MZ, SS, BD, and AP performed the experiments. MZ, WS, SS, BD, and AP analyzed the data. MZ and IM wrote the manuscript. All authors contributed to the article and approved the submitted version.

## Funding

This study was supported by the National Cancer Research Resources and the National Center for Advancing Translational Sciences, National Institute of Health, through Grant UL1 TR001414. The content is solely the responsibility of the authors and does not necessarily represent the official views of the National Institutes of Health.

## Conflict of Interest

The authors declare that the research was conducted in the absence of any commercial or financial relationships that could be construed as a potential conflict of interest.

## Publisher’s Note

All claims expressed in this article are solely those of the authors and do not necessarily represent those of their affiliated organizations, or those of the publisher, the editors and the reviewers. Any product that may be evaluated in this article, or claim that may be made by its manufacturer, is not guaranteed or endorsed by the publisher.

## Supplementary Material

Supplementary Figure 1 Dysregulation of innate immune responses in young and aged COVID-19 patients. **(A)** Levels of circulating soluble immune mediators in young and aged COVID-19 patients compared to healthy donors. **(B)** Levels of circulating soluble immune mediators in lean, overweight and obese patients in young and aged patient groups. **(C)** Flow cytometry gating strategy used to identify innate immune cells. **(D)** Linear regression analysis of the frequency of NK cell subsets with BMI in young and aged COVID-19 patients. **(E)** Representative histograms of the expression of various markers on monocytes and DC subsets. **(F)** Representative flow cytometry plots of the secretion of pro-inflammatory cytokine (IL-6 and TNF-α) by monocytes and dendritic cell subsets (mDC and pDC) in response to a cocktail of bacterial agonists. #p < 0.12, *p < 0.05, **p < 0.01, ***p < 001 and ****p < 0001.

Supplementary Figure 2 Proliferation and cytokine responses of B and T cell subsets from young and aged COVID-19 patients. **(A)** Linear regression analysis of the frequency of B cell subsets with BMI in young and aged COVID-19 patients. **(B)** Linear regression analysis of the frequency of proliferating B cell subsets with BMI in young and aged COVID-19 patients. **(C)** SARS-CoV-2-specific IgG endpoint titers in lean, overweight and obese individuals in both young and aged patient groups. **(D)** Linear regression analysis of IgG end-point titers against the receptor-binding domain (RBD) of SARS-CoV-2 spike protein with patient BMI in young and aged patients. **(E)** Flow cytometry gating strategy used to identify adaptive immune cell subsets and their proliferation. **(F)** Linear regression analysis of the frequency of proliferating of CD4+ and CD8+ T cell subsets with BMI in young and aged COVID-19 patients. **(G)** Representative histograms of the cytokine secretion by anti-CD3/CD28 stimulated and unstimulated CD4+ and CD8+ T cells. #p > 0.05 and <0.12, *p < 0.05, **p < 0.01, ***p < 001 and ****p < 0001.

Supplementary Table 1 Patient characteristics and clinical data.

Supplementary Table 2 Correlation and Simple linear regression values of measured soluble immune mediators in young and aged patients.

Supplementary Table 3 Correlation and Simple linear regression values of innate immunity in young and aged patients.

Supplementary Table 4 Correlation and Simple linear regression values of the frequency of myeloid cell subsets in young and aged patients.

Supplementary Table 5 Correlation and Simple linear regression values of the frequency of myeloid cell subsets secreting both TNF-α and IL-6 in response to a cocktail of bacterial agonists in young and aged patients.

Supplementary Table 6 Correlation and Simple linear regression values of the frequency and proliferation of the adaptive immune cell subsets in young and aged patients.

Supplementary Table 7 Correlation and Simple linear regression values of humoral immunity against SARS-CoV-2.

Supplementary Table 8 Correlation and Simple linear regression values of the frequency of CD4+ and CD8+ T cells secreting various cytokines in response to anti-CD3/CD28 stimulation in young and aged patients.
