# Supplementary figures and images for: Obesity Correlates With Pronounced Aberrant Innate Immune Responses in Hospitalized Aged COVID-19 Patients
Source: Front Immunol. 2021 Oct 11;12:760288. doi: 10.3389/fimmu.2021.760288 (PMC8542887; doi:10.3389/fimmu.2021.760288)

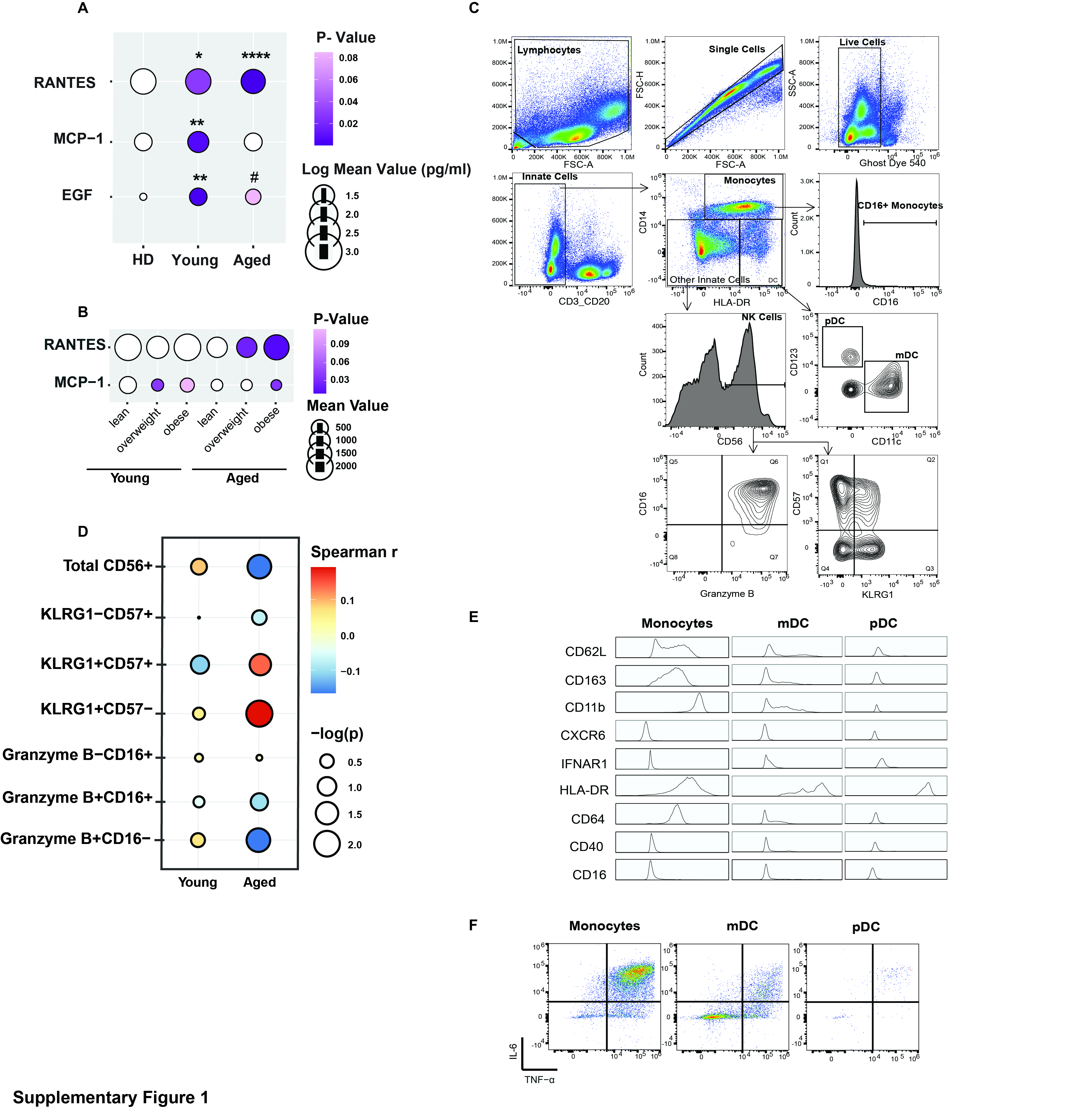

Supplement: Supplementary Figure 1 — Dysregulation of innate immune responses in young and aged COVID-19 patients. (A) Levels of circulating soluble immune mediators in young and aged COVID-19 patients compared to healthy donors. (B) Levels of circulating soluble immune mediators in lean, overweight and obese patients in young and aged patient groups. (C) Flow cytometry gating strategy used to identify innate immune cells. (D) Linear regression analysis of the frequency of NK cell subsets with BMI in young and aged COVID-19 patients. (E) Representative histograms of the expression of various markers on monocytes and DC subsets. (F) Representative flow cytometry plots of the secretion of pro-inflammatory cytokine (IL-6 and TNF-α) by monocytes and dendritic cell subsets (mDC and pDC) in response to a cocktail of bacterial agonists. #p < 0.12, *p < 0.05, **p < 0.01, ***p < 001 and ****p < 0001. [file Image_1.tif]

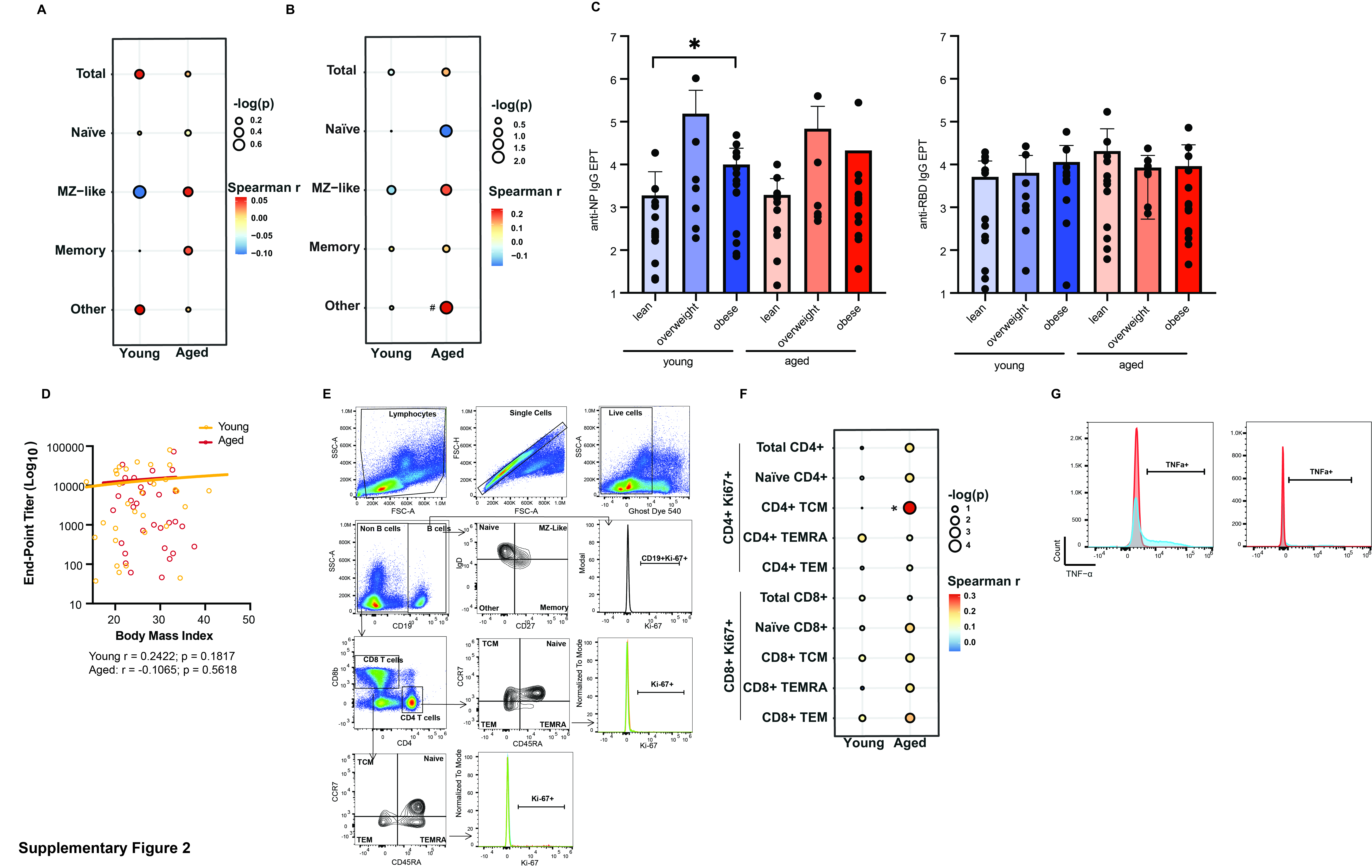

Supplement: Supplementary Figure 2 — Proliferation and cytokine responses of B and T cell subsets from young and aged COVID-19 patients. (A) Linear regression analysis of the frequency of B cell subsets with BMI in young and aged COVID-19 patients. (B) Linear regression analysis of the frequency of proliferating B cell subsets with BMI in young and aged COVID-19 patients. (C) SARS-CoV-2-specific IgG endpoint titers in lean, overweight and obese individuals in both young and aged patient groups. (D) Linear regression analysis of IgG end-point titers against the receptor-binding domain (RBD) of SARS-CoV-2 spike protein with patient BMI in young and aged patients. (E) Flow cytometry gating strategy used to identify adaptive immune cell subsets and their proliferation. (F) Linear regression analysis of the frequency of proliferating of CD4+ and CD8+ T cell subsets with BMI in young and aged COVID-19 patients. (G) Representative histograms of the cytokine secretion by anti-CD3/CD28 stimulated and unstimulated CD4+ and CD8+ T cells. #p > 0.05 and <0.12, *p < 0.05, **p < 0.01, ***p < 001 and ****p < 0001. [file Image_2.tif]
