# Supplementary material for: Obesity Correlates With Pronounced Aberrant Innate Immune Responses in Hospitalized Aged COVID-19 Patients
Source: Front Immunol. 2021 Oct 11;12:760288. doi: 10.3389/fimmu.2021.760288 (PMC8542887; doi:10.3389/fimmu.2021.760288)
Supplement: Supplementary Table 1 — Patient characteristics and clinical data. [file Table_1.docx]

**Supplementary Table 1:**

| **Patrient ID** | **Sex** | **Age** | **BMI** | **DPSO*** | **Group** | **COVID-19 Severity** |
| --- | --- | --- | --- | --- | --- | --- |
| COV001 | M | 39 | 16.94 |  | Young | ICU |
| COV002 | M | 28 | 19.64 |  | Young | Ward |
| COV003 | M | 57 | 21.99 | 3 | Young | ICU |
| COV004 | M | 19 | 22.41 |  | Young | Ward |
| COV005 | M | 25 | 22.66 |  | Young | Death |
| COV006 | M | 25 | 22.66 |  | Young | ICU |
| COV007 | M | 59 | 22.73 |  | Young | Ward |
| COV008 | M | 59 | 22.98 | 15 | Young | ICU |
| COV009 | M | 59 | 23.53 | 11 | Young | ICU/I |
| COV010 | M | 25 | 23.56 | 3 | Young | Ward |
| COV011 | M | 40 | 23.6 | 67 | Young | ICU/I |
| COV012 | M | 49 | 25.24 | 22 | Young | ICU |
| COV013 | M | 54 | 25.24 | 30 | Young | ICU/I |
| COV014 | M | 56 | 25.66 |  | Young | ICU |
| COV015 | M | 43 | 26.3 |  | Young | Ward |
| COV016 | M | 53 | 26.52 | 10 | Young | Ward |
| COV017 | M | 30 | 27.38 | 18 | Young | ICU/I |
| COV018 | M | 42 | 27.99 | 28 | Young | ICU |
| COV019 | M | 50 | 28.08 | 16 | Young | Ward |
| COV020 | F | 42 | 28.76 | 55 | Young | ICU/I |
| COV021 | M | 51 | 29.33 |  | Young | ICU |
| COV022 | F | 51 | 29.43 | 8 | Young | Ward |
| COV023 | F | 53 | 29.86 | 1 | Young | ER |
| COV024 | M | 59 | 53 | 36 | Young | Death |
| COV025 | F | 54 | 35.67 | 37 | Young | Death |
| COV026 | M | 57 | 35.95 | 46 | Young | Death |
| COV027 | M | 54 | 35.67 | 72 | Young | Death |
| COV028 | F | 59 | 37.65 |  | Young | Ward |
| COV029 | F | 32 | 34.42 | 4 | Young | Ward |
| COV030 | F | 30 | 45.53 |  | Young | Death |
| COV031 | F | 59 | 34.92 | 5 | Young | Ward |
| COV032 | F | 46 | 32.02 | 39 | Young | Death |
| COV033 | M | 55 | 31.32 |  | Young | Ward |
| COV034 | M | 42 | 35.11 | 8 | Young | Ward |
| COV035 | F | 51 | 39.53 | 25 | Young | Ward |
| COV036 | M | 54 | 38.45 | 18 | Young | Death |
| COV037 | F | 41 | 31.26 | 13 | Young | ICU |
| COV038 | F | 55 | 35.93 | 26 | Young | ICU/I |
| COV039 | F | 23 | 34.45 |  | Young | ICU/I |
| COV040 | F | 78 | 18.43 |  | Aged | ICU |
| COV041 | M | 90 | 19.53 | 14 | Aged | Death |
| COV042 | F | 85 | 20.7 | 50 | Aged | Death |
| COV043 | M | 73 | 21.41 | 1 | Aged | ER |
| COV044 | M | 74 | 21.6 |  | Aged | Ward |
| COV045 | M | 67 | 21.63 | 29 | Aged | ICU |
| COV046 | M | 76 | 22.23 |  | Aged | ICU/I |
| COV047 | M | 68 | 22.86 | 24 | Aged | ICU/I |
| COV048 | M | 69 | 23.15 | 11 | Aged | Ward |
| COV049 | M | 84 | 23.4 | 6 | Aged | Ward |
| COV050 | M | 82 | 23.51 | 34 | Aged | Death |
| COV051 | M | 77 | 23.84 |  | Aged | Ward |
| COV052 | M | 67 | 24.19 |  | Aged | Ward |
| COV053 | F | 68 | 24.61 | 14 | Aged | Ward |
| COV054 | F | 92 | 24.75 | 7 | Aged | Ward |
| COV055 | F | 92 | 29.38 | 47 | Aged | Death |
| COV056 | M | 67 | 26.66 | 47 | Aged | ICU/I |
| COV057 | M | 73 | 28.69 | 38 | Aged | Death |
| COV058 | F | 68 | 27.18 | 16 | Aged | Ward |
| COV059 | F | 62 | 26.37 | 31 | Aged | Death |
| COV060 | M | 64 | 26.82 | 17 | Aged | Ward |
| COV061 | M | 81 | 29.75 | 8 | Aged | Death |
| COV062 | M | 73 | 25.7 | 5 | Aged | Ward |
| COV063 | M | 89 | 26.18 | 8 | Aged | ICU/I |
| COV064 | F | 70 | 28.6 | 25 | Aged | Death |
| COV065 | F | 67 | 29.73 |  | Aged | ICU/I |
| COV066 | M | 68 | 28.45 |  | Aged | Death |
| COV067 | M | 84 | 28.28 | 9 | Aged | Ward |
| COV068 | M | 60 | 26.82 | 35 | Aged | Ward |
| COV069 | M | 84 | 28.76 | 26 | Aged | Ward |
| COV070 | M | 63 | 26.46 | 7 | Aged | ICU/I |
| COV071 | M | 82 | 25.96 | 7 | Aged | Ward |
| COV072 | M | 63 | 26.95 | 24 | Aged | Death |
| COV073 | F | 62 | 26.37 | 68 | Aged | Ward |
| COV074 | F | 68 | 28.1 |  | Aged | Ward |
| COV075 | M | 66 | 27.43 | 58 | Aged | ICU/I |
| COV076 | M | 71 | 26.41 |  | Aged | Ward |
| COV077 | M | 65 | 26.16 | 35 | Aged | Death |
| COV078 | M | 64 | 29.27 | 11 | Aged | Ward |
| COV079 | M | 81 | 26.41 | 20 | Aged | Death |
| COV080 | M | 76 | 27.8 |  | Aged | Ward |
| COV081 | M | 85 | 25.4 | 21 | Aged | Ward |
| COV082 | F | 77 | 28.9 | 12 | Aged | Ward |
| COV083 | F | 74 | 32.73 | 13 | Aged | Death |
| COV084 | F | 75 | 32.85 | 14 | Aged | Death |
| COV085 | M | 61 | 33.01 | 56 | Aged | Death |
| COV086 | M | 76 | 33.42 | 22 | Aged | Ward |
| COV087 | M | 70 | 34.9 | 24 | Aged | ICU |

***Days post symptoms onset**
